# Fluorescence *in situ* hybridization (FISH) and cell sorting of living bacteria

**DOI:** 10.1038/s41598-019-55049-2

**Published:** 2019-12-09

**Authors:** Giampiero Batani, Kristina Bayer, Julia Böge, Ute Hentschel, Torsten Thomas

**Affiliations:** 1 Centre for Marine Science and Innovation and School of Biological, Earth and Environmental Sciences, The University of New South Wales, Sydney, New South Wales Australia; 2 Faculty of Science - Department of Parasitology, University of South Bohemia, Ceske Budejovice, Czech Republic; 3 GEOMAR Helmholtz Centre for Ocean Research Kiel, Düsternbrooker Weg 20, 24105 Kiel, Germany; 4 Christian-Albrechts University of Kiel, Christian-Albrechts-Platz 4, 24118 Kiel, Germany

**Keywords:** Applied microbiology, Microbial ecology

## Abstract

Despite the development of several cultivation methods, the rate of discovery of microorganisms that are yet-to-be cultivated outpaces the rate of isolating and cultivating novel species in the laboratory. Furthermore, no current cultivation technique is capable of selectively isolating and cultivating specific bacterial taxa or phylogenetic groups independently of morphological or physiological properties. Here, we developed a new method to isolate living bacteria solely based on their 16S rRNA gene sequence. We showed that bacteria can survive a modified version of the standard fluorescence *in situ* hybridization (FISH) procedure, in which fixation is omitted and other factors, such as centrifugation and buffers, are optimized. We also demonstrated that labelled DNA probes can be introduced into living bacterial cells by means of chemical transformation and that specific hybridization occurs. This new method, which we call live-FISH, was then combined with fluorescence-activated cell sorting (FACS) to sort specific taxonomic groups of bacteria from a mock and natural bacterial communities and subsequently culture them. Live-FISH represents the first attempt to systematically optimize conditions known to affect cell viability during FISH and then to sort bacterial cells surviving the procedure. No sophisticated probe design is required, making live-FISH a straightforward method to be potentially used in combination with other single-cell techniques and for the isolation and cultivation of new microorganisms.

## Introduction

While the “-omics” era has greatly increased our knowledge on the identity and putative function of yet-to-be-cultivated microorganisms, cultivation still remains an important tool to obtain isolates for physiological characterization or to recover natural products of interest^1–3^. However, cultivation is challenging, as it is often difficult to replicate the specific and essential aspects of a microorganism’s environment that would lead to a selective and successful growth in the laboratory^4^. Several methods have been developed with the aim to selectively culture certain microorganisms or groups of microorganisms^4^. For example, dilution-to-extinction culturing has been successfully used to cultivate the most abundant organisms in a sample, even with non-selective media, as exemplified by the cultivation of SAR11 clade bacteria (e.g. *Pelagibacter ubique*) from seawater^5^. This method does however not work for less abundant microorganisms in a sample. Micromanipulators^6^ or laser manipulation systems, such as optical tweezers^7^, can also be used to isolate specific bacteria, as demonstrated by the plant pathogen *Leifsonia xyli*^8^ or the archaeal genera *Thermofilum* and *Thermoproteus*^9^. These approaches however require unique morphological features that can be used to identify the targeted organism. Finally, fluorescence-activated cell sorting (FACS) has been used to sort cells based on inherent fluorescence or morphological properties for subsequent cultivation^10–12^. However, none of these techniques is capable of selectively isolating specific bacterial taxa or phylogenetic groups independently of their morphological or physiological properties or their abundance in a sample.

A powerful and widely used approach for targeting specific groups of microorganisms is fluorescence *in situ* hybridization (FISH), where labelled DNA probes are used to target rRNA of defined taxonomic or phylogenetic groups^13,14^. Standard FISH protocols employ chemical cross-linking (or fixation), typically with paraformaldehyde, to stabilize the cells as well as partial cell wall lysis, often involving ethanol, to allow for probe penetration^15–17^. These steps result in chemical modification of nucleic acids as well as cell death. Recently, fixation-free FISH (FFF)^18,19^ has been developed to avoid complications with DNA extraction due to the chemical cross-linking. The FFF protocol still employs an ethanol step to make the cells permeable for the probes^19^. However, it is well known that DNA probes can be introduced with high efficiencies into living bacterial cells via different processes, such as natural and chemical transformation or electroporation^20^. The possibility of using one of these transformation techniques instead of an ethanol treatment to deliver fluorescent probes into living bacteria remains however largely unexplored. The only study we are aware of using fluorescent probe hybridization in living bacteria is by Silverman and Kool^21^, who used a small amount of detergent (0.05% sodium dodecyl sulfate, SDS) to “soften” the bacterial cell wall and to introduce the highly specific, quenched autoligation (QUAL) probes^22^. However, there has been a controversy whether the hybridized cells were really alive, as live/dead staining showed that the treated cell suspensions were heterogeneous and comprised mainly of dead cells^23^. Moreover, treatments with 0.05% SDS have been reported to kill the majority of *Escherichia coli* cells in suspensions^23^. Nevertheless, probe hybridization in living cells has been reported for a number of eukaryotic cell types^24^, which indicates that there may be no inherent biological limitation for live hybridization also working with bacteria if probes can be delivered without killing the cells.

In this work, we aimed to develop a new method for the isolation of specific living bacteria based on a) fluorescent labelling bacteria with DNA probes without killing them, b) the specific isolation of these labelled cells using FACS and c) cultivation of these labelled and sorted cells on non-selective media. We call the developed protocol live-FISH and showed that, when used in combination with FACS, allows for the isolation of Gram-positive and Gram-negative living bacteria that belong to certain taxonomic groups as defined by the probe target.

## Material and Methods

### Bacterial cultures and sample preparation

The strains used in this study were *Bacillus* sp. AU29 (phylum Firmicutes)^25^, *Ruegeria* sp. AU82 (order Rhodobacterales, class Alphaproteobacteria)^25^, *Pseudovibrio* sp. SB55 (order Rhodobacterales, class Alphaproteobacteria)^26^ and *Amphritea atlantica* M41^T^ (order Oceanospirillales, class Gammaproteobacteria)^27^ and were provided by the authors of the cited references. Cells were grown in Marine Broth (MB) medium (Difco 2216, BD Biosciences, San Jose, USA) at 25 °C with shaking at 200 rpm and harvested during late logarithmic growth phase (OD_600nm_ = 0.5–0.8). Aliquots containing 20% glycerol were then stored at −80 °C. In order to perform further analyses on living cells, stock cultures were slowly thawed on ice, inoculated in fresh MB (1:100) and grown again to late logarithmic phase. Baltic surface seawater (54.329737°N, 10.149379°E) was sampled in triplicates during May 2018 and pre-filtered through 50 µm syringe filcons (BD Biosciences, San Jose, USA). To concentrate seawater bacteria to ~10^8^ cells ml^−1^, 5L of pre-filtered seawater were further filtered through a 0.2 µm Zeta Plus 1MDS positively charged filters (CUNO Incorporated, Meriden, USA) and the adsorbed bacteria were eluted by passing 1 ml of MB in the direction opposite to the influent flow^28^. These aliquots were then pelleted, resuspended in 1 ml of pre-filtered seawater and stored briefly on ice before using in the experiments below.

### FISH probes

Table 1 lists the FISH probes and the hybridization conditions used in this study. Probes were labelled with 6-carboxyfluorescein (6-FAM - a derivative of fluorescein-isothiocyanate (FITC)) or cyanine 3 (Cy3). The specificity of the probes was confirmed by BLASTn searches against the NT database at the National Centre of Biotechnology Information (NCBI). The required stringency of the hybridization conditions was pre-evaluated *in silico* using mathFISH^29^ and confirmed using formamide gradients. The sequences of the probes LGC399 (targeting the phylum Firmicutes), PARA739 (targeting the order Rhodobacterales) and ALF968 (targeting the class Alphaproteobacteria, except for Rickettsiales) were obtained from probeBase^30^ and used to detect *Bacillus* sp. AU29 (probe LGC399), *Ruegeria* sp. AU82 and *Pseudovibrio* sp. SB55 (probe PARA739) and Alphaproteobacteria (probe ALF968). The universal bacterial probe EUB338 was used as a positive control. The NONEUB338 probe was used as a negative control.

**Table 1:** Fluorescence *in situ* hybridization probes and conditions used in this study.

Name	Label	Sequence (5′–3′)	Target	Formamide (%)	[NaCl] (M)	Reference
LGC399	6-FAM*	TCACGCGGCGTTGCTC	Firmicutes	35	0.080	Küsel *et al*. (1999)
PARA739	6-FAM	GCGTCAGTATCGAGCCAG	Rhodobacterales	35	0.080	Thayanukul *et al*. (2010)
ALF968	6-FAM	GGTAAGGTTCTGCGCGTT	Alphaproteobacteria, except Rickettsiales	25	0.159	Alm *et al*. (1996)
EUB338	Cy3	GCTGCCTCCCGTAGGAGT	Most bacteria	35	0.080	Amann *et al*. (1990)
NONEUB338	6-FAM or Cy3	ACTCCTACGGGAGGCAGC	—	35	0.080	Wallner *et al*. (1993)

*6-Carboxyfluorescein.

### Modification of the FFF protocol and assessment of bacterial viability

In order to assess whether cells were still alive or not after the previously described FFF protocol^19^, colony-forming units (CFU) were determined on MB agar plates after each protocol step. After initially growing an overnight culture, cells were washed three times with 1x Phosphate Buffered Saline solution (PBS: 137 mM NaCl, 2.7 mM KCl, 10 mM Na_2_HPO_4_, 1.8 mM KH_2_PO_4_) rather than washing with an ethanol series as performed in the FFF protocol by Haroon *et al*.^19^, which would have killed the cells. In addition, we introduced a heat shock step after the washing, which was performed according to Froger and Hall^31^ to facilitate nucleic acid uptake. Briefly, washed cells were first resuspended in 50 µl of 100 mM CaCl_2_, then incubated for 15 min on ice with 4 ng µl^−1^ of the fluorescent probe (500 ng µl^−1^, Biomers, Germany), further incubated at 42 °C for 60 sec (heat shock) and then briefly placed back on ice. All incubations were performed with shaking at 200 rpm. 500 µl of pre-warmed (46 °C) hybridization buffer (0.9 M NaCl, 20 mM Tris-HCl pH 7.4, 0.01% SDS, 35% formamide) were immediately added and hybridization was carried out by incubation for 2 h at 46 °C. After hybridization, cells were pelleted at 10,000 × g for 5 min and washing was performed as follows: samples were first resuspended in 500 µl of pre-warmed (48 °C) wash buffer (20 mM Tris-HCl, 5 mM EDTA, 0.01% SDS, 0.080M NaCl), pelleted at 10,000 × g for 5 min, resuspended again in 500 µl of wash buffer and incubated at 48 °C for 20 min. Finally, cells were centrifuged twice in 500 µl of ice-cold 1x PBS and kept in this buffer on ice until further analyses.

### Optimization of the live-FISH procedure

The final developed protocol for FISH on living bacteria (live-FISH) is shown in Table 2. The procedure was optimized using the Gram-positive *Bacillus* sp. AU29 and the Gram-negative bacteria *Ruegeria* sp. AU82 and *Pseudovibrio* sp. SB55 with different resuspension buffers, centrifugation speeds and incubation times (Supplementary Fig. S1). For the pre-hybridization process, combinations of three different buffers (PBS; artificial seawater (ASW): 400 mM NaCl, 10 mM KCl, 19.9 mM MgCl_2_, 20 mM MgSO_4_, 13.2 mM CaCl_2_, 2 mM NaHCO_3_; Ca^2+^- and Mg^2+^-free artificial seawater (CMFASW): 430 mM NaCl, 10 mM KCl, 7 mM Na_2_SO_4_, 0.5 mM NaHCO_3_), two centrifugation speeds (13,000 × g and 10,000 × g) and two incubation times (10 min and 5 min) were tested. Buffers that could mimic the natural environment of marine bacteria are likely important to increase their viability, along with any effect that centrifugation speed and length could have on bacterial recovery. The heat shock step was optimized by a combination of incubations in different solutions. Specifically, bacteria were resuspended in a cold solution of MgCl_2_/CaCl_2_ at different molarities (either 80 mM MgCl_2_/20 mM CaCl_2_ or 160 mM MgCl_2_/40 mM CaCl_2_) in order to preserve the cell membrane by getting close to the seawater osmolarity (~1000 mOsm/l), before incubation in 50 µl of 100 mM CaCl_2_ with the probe on ice for 15 min and at 42 °C for 60 sec (heat shock). It is well established that cations are a key factor in the transformation process^32,33^, with Mg^2+^ and Ca^2+^ showing a strong effect on transformation of *Escherichia coli* cells^32^. The cations’ positive charge may help to condensate the free DNA by shielding the negative phosphate groups, hence making it small enough for cellular uptake. Cations can also cancel out electrostatic repulsion between DNA and the outer membrane and thereby facilitate DNA-membrane contacts^34^. Bacterial viability was also tested as a function of hybridization time (1, 1.5, 2, 2.5 and 3 h). Finally, different fluorophores (Cy3 vs 6-FAM), wash buffer volumes (500 µl vs 1 ml) and centrifugation/incubation times (5 min centrifugation plus 20 min incubation in wash buffer versus 2 × 15 min centrifugation and no incubation) were tried in order to avoid unwanted background or unspecific signals detected during flow cytometry analyses. Statistical comparisons among the different treatments were performed using the non-parametric Kruskal-Wallis test, followed by Dunn’s multiple comparisons test. The change of viability over time during optimization of the hybridization step was assessed by linear regression analysis (GRAPHPAD PRISM version 7.03 for Windows; GRAPHPAD Software).

**Table 2:** Summary of steps for live-FISH procedure.

Stage	Step no.	Description
Pre-hybridization	1	Mix 1 ml of overnight culture with 500 µl CMFASW^a^.
	2	Centrifuge at 13,000 × g for 5 min at RT^b^.
	3	Wash twice in 500 µl CMFASWa at 13,000 × g for 5 min at RTb.
Heat shock	4	Resuspend pellets in 1 ml ice cold 160 mM MgCl_2_/40 mM CaCl_2_.
	5	Centrifuge at 13,000 × g for 5 min at 4 °C.
	6	Resuspend pellets in 50 µl ice cold 100 mM CaCl_2_ + 4 µl probe (500 ng/µl).
	7	Incubate 15 min on ice in the dark.
	8	Incubate for 60 sec at 42 °C.
Hybridization	9	Add immediately 500 µl of pre-warmed hybridization buffer.
	10	Incubate at 46 °C in the dark for 2 hrs.
Washing	11	Centrifuge at 13,000 × g for 5 min at RT^b^.
	12	Wash twice in 1 ml pre-warmed wash buffer at 13,000 × g for 15 min at 48 °C.
	13	Re-suspend in 500 µl CMFASW^a^ and keep on ice in the dark until sorting.

^a^CMFASW, Ca^2+^- and Mg^2+^-free artificial seawater.

^b^RT, room temperature.

### Microscopy

Cells were visualized with an Axio Observer.Z1 microscope equipped with AxioCam 506 and Zen 2 version 2.0.0.0 software (Carl Zeiss Microscopy GmbH, Göttingen, Germany) and counterstained using the live/dead BacLight bacterial viability kit L7007 (ThermoFisher Scientific, Germany) containing SYTO9 dye and propidium iodide (PI) for the detection of living and dead cells, respectively. PI cannot cross the membrane of living cells and hence only stains intracellular nucleic acids when the cell membrane is compromised^35^. SYTO9 can readily penetrate the cell membrane to bind to nucleic acids, but gets displaced from the nucleic acids and quenched when PI is in the cell^36^. Optical microscope filters were selected according to the fluorochromes used (max. excitation/emission in nm: SYTO9 485/498; 6-FAM 490/525, propidium iodide 535/617).

### Cell sorting

Immediately after live-FISH and before sorting, all samples were filtered using 50 µm syringe filters (BD Biosciences) to remove aggregates and stained with 0.05 µg ml^−1^ PI (Sigma-Aldrich, Germany) in order to distinguish the dead cells from those that are living and hybridized. Cell sorting experiments were performed on a MoFlo®Astrios EQ^TM^ High Speed Sorter (Beckman Coulter, Indianapolis, USA) using the sheath fluid 1x PuraFlo^TM^, which had a ~50% survival rate when bacteria were incubated in it for 48 h (data not shown). In a typical analysis run, between 100,000 to 250,000 events were recorded. A LD laser with a wavelength of 488 nm was used at a power of up to 100 mW. The side-scattered light (SSC) signal was detected by using the 488 nm laser and used as trigger parameter. The 6-FAM and PI signals were detected by using the 526/52 and 620/29 or 664/22 nm filters (all 488 nm laser), respectively. SPHERO^TM^ Ultra Rainbow Fluorescent Particles (Spherotech Inc., Lake Forest, Il, USA) were used to establish appropriate instrument settings for the daily set- up and the threshold for the minimal detectable fluorescence intensity was set up between 10^2^ and 10^3^ arbitrary units (a.u.). Data were analyzed using the Summit software version 6.3.0.16900 (Beckman Coulter, Brea, USA) and the percentages of the sorted populations were calculated with respect to the total cell counts.

Cells were sorted into 96-well plates filled with 200 µl MB per well. One to three columns per plate were always used as a negative control for contamination (i.e. no cells were sorted). Batches of 1, 10 or 100 target cells were sorted. All plates were incubated at 25 °C with breathable sealing mats.

Sorted cells were identified by colony PCR and sequencing of the 16S rRNA gene according to Costa and Weiner^37^, with minor modifications. Briefly, colonies were gently stabbed with a sterile pipette tip and swirled into a reaction tube containing 50 µl of the PCR master mix (1x DreamTaq Green Buffer (ThermoFisher Scientific, Germany), 2 mM of each primer (Sigma Aldrich, Germany), 0.2 mM dNTPs (ThermoFisher Scientific, Germany) and 1.25 U DreamTaq Polymerase (ThermoFisher Scientific). Amplification of the 16S rRNA genes was done with a Biometra TRIO Combi Thermocycler (Analytik Jena, Germany) and the universal primer pairs 27F and 1492R^38^. PCR conditions were as follows: 5 min at 98 °C followed by 30 sec at 98 °C, 30 sec at 55 °C, 1.5 min at 72 °C for 30 cycles and a final step of 72 °C for 5 min. One microliter of *A. atlantica* genomic DNA (100 ng) and 1 µl of diethyl pyrocarbonate -treated water were used as positive and negative controls, respectively. The resulting PCR products were analyzed on 1.5% agarose gels. Positive products were purified using the NucleoSpin Gel and PCR Clean-up kit (Machery-Nagel GmbH, Germany) and sequenced on a LightRunNXP tube platform (EUROFINS, Germany). The identity of the cells was assessed by BLASTn searches of the ~1200 bp sequences against the NT database.

All experiments were performed at least in duplicates. Cell growth is presented as averages with standard deviations of these experiments, while one representative density scatterblot plot is being shown.

To optimize the sorting protocol, *Bacillus* sp. AU29 and *Pseudovibrio* sp. SB55 were first sorted separately. Subsequently, *Bacillus* sp. AU29, *Pseudovibrio* sp. SB55 and *A. atlantica* M41^T^ were mixed 1:1:1 and live-FISH and cell sorting were carried out on this mock community with *Pseudovibrio* sp. SB55 being the target bacterium. *Pseudovibrio* sp. SB55 was also inoculated 1:1 into Baltic seawater after diluting it to 10^6^ cells ml^−1^ (which was the bacterial concentration of the Baltic seawater measured by flow cytometry; data not shown), and live-FISH was carried out on the whole sample to test whether it was possible to isolate FISH-labelled *Pseudovibrio* sp. SB55 cells from the seawater microbial community. Finally, the protocol was applied directly to the seawater to sort bacteria of interest from a natural community. For this experiment, a probe ALF968 targeting Alphaproteobacteria was used, as this group is typically present in the bacterial community of Baltic seawater^39^.

Two negative controls were used for all experiments, one without adding any probe (“no probe” control), and the other being a “non-target” probe, which means that *Bacillus* sp. AU29 cells were treated with a probe for *Pseudovibrio* sp. SB55, and vice versa. The “non-target” probes had more than 3 mismatches to the non-target sequences. For the mock community and the seawater experiments, the NONEUB338 probe was used as a negative “non-sense” control (Table 1).

## Results

### Recovery and visualization of living cells after FFF

**Figure 1:**
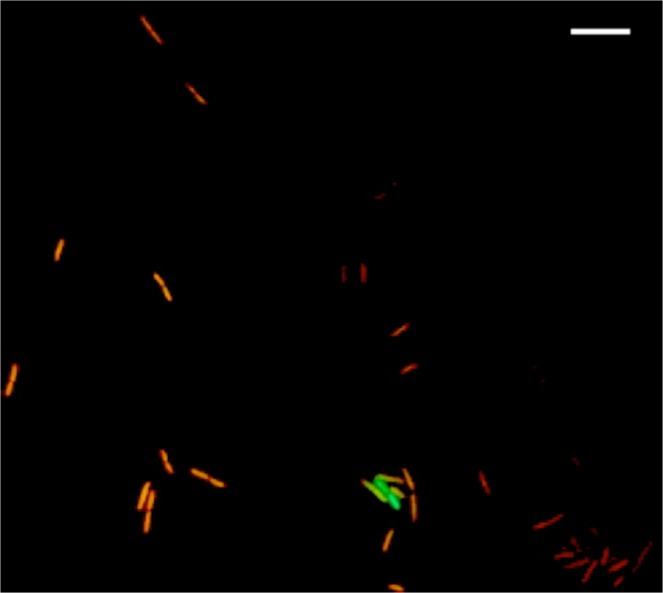
Pure culture of *Bacillus* sp. AU29 after FFF (EUB338_Cy3 probe) and counterstained with the live/dead kit. Red cells are propidium iodide (PI) positive and hence dead, green cells are SYTO 9 positive and hence alive, yellow cells are FISH and SYTO9 positive and hence both living and containing the probe. Scale bar, 5 µm.

The viability of *Bacillus* sp. AU29 and *Ruegeria* sp. AU82 was assessed at all steps of a FFF protocol^19^, where the ethanol dehydration step was replaced by a PBS wash and a heat shock was applied^31^ in an effort to improve cell survival and facilitate DNA uptake, respectively. We found that 0.10% of *Bacillus* sp. AU29 cells and 0.07% of *Ruegeria* sp. AU82 cells did survive the procedure (Table 3). To investigate if some of these viable cells were also FISH-labelled, live/dead counterstaining and fluorescence microscopy were performed on *Bacillus* sp. AU29 processed with the modified FFF protocol using the EUB338_Cy3 probe. This revealed the occasional presence of yellow cells derived from the combination of Cy3 (FISH probe) with SYTO9 (live stain) signals and indicated that indeed some cells were both alive and contained the probe (Fig. 1).

**Table 3:** CFU ml^−1^ values at all steps of the modified FFF protocol and percentage viability (in brackets) with respect to values after overnight incubation (control) for both *Bacillus* sp.

FISH steps	*Bacillus* sp. AU29 CFU ml^−1^(%)	*Ruegeria* sp. AU82 CFU ml^−1^(%)
Control	5.80E + 08	3.70E + 08
Pre-hybridization	7.20E + 07 (12.41)	5.20E + 07 (14.05)
Heat shock	3.07E + 07 (5.30)	1.07E + 07 (2.90)
Hybridization	8.00E + 06 (1.38)	9.80E + 06 (2.65)
Washing	5.73E + 05 (0.10)	2.73E + 05 (0.07)

AU29 and *Ruegeria* sp. AU82.

### Optimization of the FISH steps to improve bacterial viability

After these initial observations, we next aimed to increase viability of cells during the procedure. Combinations of different buffers, centrifugation speeds and incubation times were tested (Supplementary Fig. S1) in order to recover more viable cells when compared to the modified FFF protocol used above (PBS - 10,000 × g - 5 min, see Supplementary Table S1). The only significant increase in cell viability was for treatment with CMFASW - 13,000 × g - 5 min for both *Bacillus* sp. AU29 (p ≤ 0.05) and *Ruegeria* sp. AU82 (p ≤ 0.05) when compared to the modified FFF process (Supplementary Table S1). There was also no statistical support that this treatment with CMFASW - 13,000 × g - 5 min was different to the cell numbers before the treatments (Supplementary Table S2), with only 31.94% cell loss for *Bacillus* sp. AU29 (p > 0.1) and 28.07% cell loss for *Ruegeria* sp. AU82 (p > 0.1) (Table 4), compared to 87.59% and 85.95%, respectively, for the modified FFF protocol (Table 3).

**Table 4:** CFU ml^−1^ values at all steps of the modified FFF protocol and percentage viability (in brackets) with respect to values after overnight incubation (control) for *Bacillus* sp.

FISH steps	*Bacillus* sp. AU29 CFU ml^−1^(%)	*Ruegeria* sp. AU82 CFU ml^−1^(%)	*Pseudovibrio* sp. SB55 CFU ml^−1^(%)
Control	7.20E + 08	5.31E + 08	6.50E + 08
Pre-hybridization	4.90E + 08 (68.06)	3.82E + 08 (71.93)	2.89E + 08 (44.46)
Heat shock	2.09E + 08 (29.05)	1.59E + 08 (29.88)	1.02E + 08 (15.69)
Hybridization	7.44E + 07 (10.34)	2.20E + 07 (4.14)	1.34E + 07 (2.06)
Washing	8.90E + 06 (1.24)	1.50E + 07 (2.82)	1.01E + 07 (1.55)

AU29, *Ruegeria* sp. AU82 and *Pseudovibrio* sp. SB55.

The conditions for the heat shock were also optimized. A significant difference in viability was observed only between the control (i.e. cell numbers before the treatments) and the modified FFF protocol (incubation in only cold 100 mM CaCl_2_) for both *Bacillus* sp. AU29 and *Pseudovibrio* sp. SB55 (p ≤ 0.05), while for the resuspension and centrifugation in a cold solution of either 80 mM MgCl_2_/20 mM CaCl_2_ or 160 mM MgCl_2_/40 mM CaCl_2_ was no significant difference to the initial cell numbers (Supplementary Table S3).

Furthermore we aimed to find the best incubation time in hybridization buffer to get viable cells while still maintaining a fluorescent signal detectable by microscopy. Viability was seen to significantly decrease over the hybridization time for both *Bacillus* sp. AU29 (F (1, 3) = 976.6, p < 0.0001, R^2^ = 0.9969) and *Ruegeria* sp. AU82 (F (1, 3) = 40.56, p = 0.0078, R^2^ = 0.9311) (Supplementary Fig. S2). Fluorescence intensity was investigated under the microscope by keeping the exposure time at a fixed value based on the NONEUB338 probe negative control. A signal in the cells was only observed from 2 h of hybridization onwards for both species analyzed (Supplementary Fig. S2). Thus, a hybridization time of 2 h was chosen as it allows probe penetration and/or hybridization, while minimizing bacterial cell loss (estimated to be 18.71% and 25.74% after the heat shock for *Bacillus* sp. AU29 and *Ruegeria* sp. AU82, respectively - Table 4).

The overall recovery of viable cells was assessed by combining all these optimization steps into a final live-FISH protocol and this showed >10-fold increase in the final viable cell numbers for both *Bacillus* sp. AU29 (t (4) = 4.248, p < 0.05) and *Ruegeria* sp. AU82 (t (4) = 3.007, p < 0.05) when compared to the initial modified FFF protocol (Tables 3 and 4). Overall >1% of the initial cells survived the new live-FISH process and this was also found to apply to *Pseudovibrio* sp. SB55 (Table 4).

### Identification and sorting of FISH-labelled and living cells

**Figure 2:**
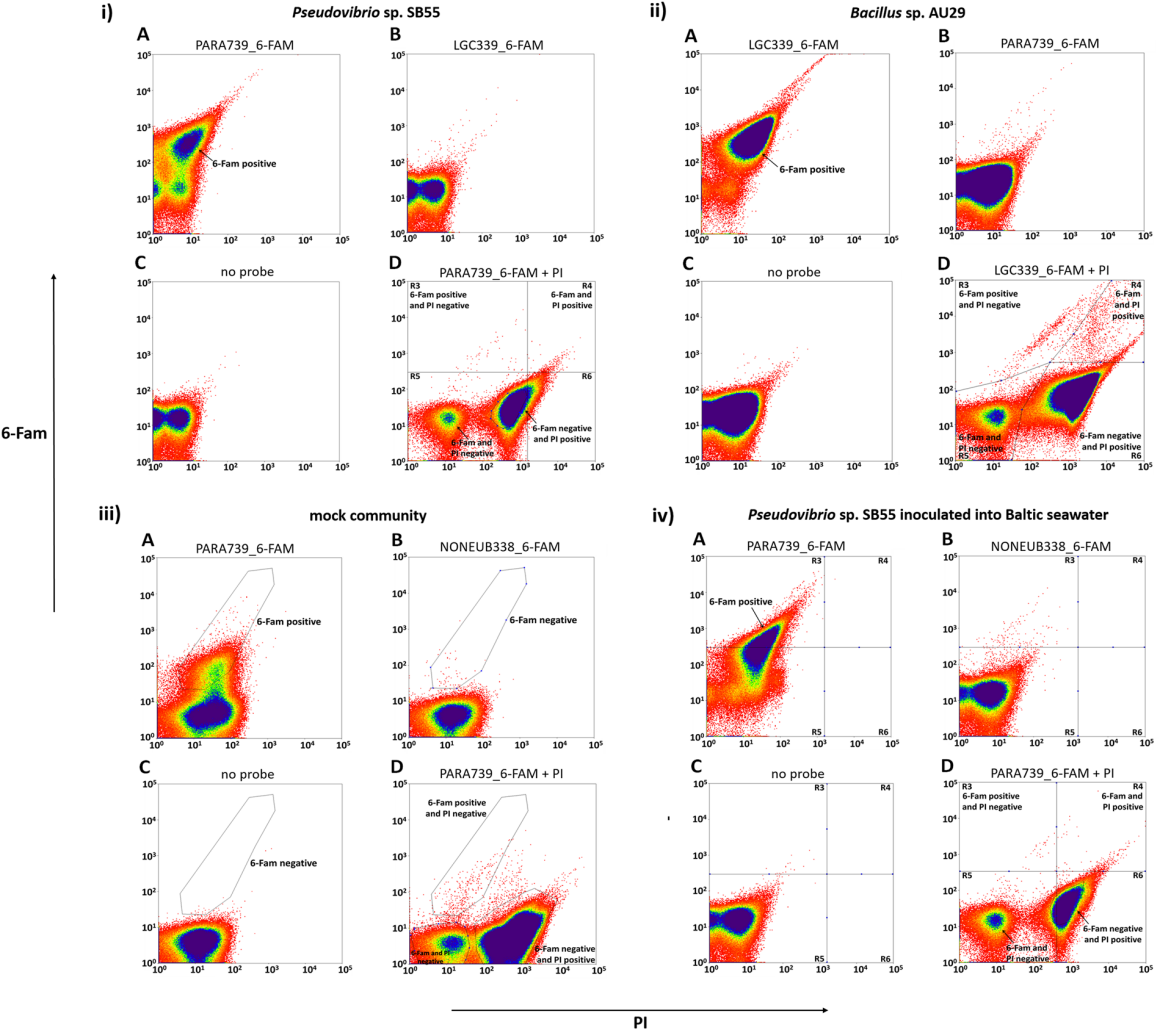
Signals for PI (X axes) and 6-FAM (Y axes) are plotted to show the sorting of (i) *Pseudovibrio* sp. SB55 when hybridized with (**A**) the probe PARA739_6-FAM, (**B**) the “non-target” LGC339_6-FAM probe, (**C**) “no probe” and (**D**) PARA739_6-FAM + PI; (ii) *Bacillus* sp. AU29 when hybridized with (**A**) the probe LGC339_6-FAM, (**B**) the “non-target” PARA739_6-Fam probe, (**C**) “no probe” and (**D**) LGC339_6-FAM + PI; (iii) the mock community when hybridized with (**A**) the probe PARA739_6-FAM for *Pseudovibrio* sp. SB55, (**B**) the “non-sense” NONEUB338_6-FAM probe, (**C**) “no probe” and (**D**) PARA739_6-FAM + PI; (iv) *Pseudovibrio* sp. SB55 inoculated into Baltic seawater when hybridized with (**A**) the probe PARA739_6-FAM, (**B**) the “non-sense” NONEUB338_6-FAM probe, (**C**) “no probe” and (**D**) PARA739_6-FAM + PI. In all D panels, either the gates or the quadrants indicate the cell populations that were distinguished after counterstaining with PI and sorted.

In order to distinguish living cells from dead ones and thus reliably sort only the living cells holding a fluorescent signal, *Pseudovibrio* sp. SB55 or *Bacillus* sp. AU29 were stained with PI at the end of the live-FISH procedure and then analyzed by FACS. For both organisms, clear probe-specific signals (6-FAM, Fig. 2iA, iiA) were observed when compared to the “non-target” probe (Fig. 2iB, 2iiB) and “no probe” (Fig. 2iC, 2iiC) controls. This shows that 6-FAM signal can only be obtained when a probe complementary to the target sequence is applied and that the simple presence of a non-specific, fluorescent probe does not result in a stable or detectable signal. This strongly suggests that the 6-FAM signal from the specific probes results from hybridization to its target.

Staining with PI showed a small, but clearly detectable population (0.02% of all event counts) of 6-FAM positive and PI negative (and hence likely living) cells for *Pseudovibrio* sp. SB55 (R3 quadrant, Fig. 2iD). 6-FAM and PI positive (hybridized, but dead) cells (R4 quadrant, Fig. 2iD) made up 0.10% of all cell counts, which indicates a survival rate of about 17% for cells that underwent successful hybridization. The majority of cells (63.42%) were however 6-FAM negative and PI positive and hence not hybridized and dead (R6 quadrant, Fig. 2iD). There was also an unstained population (both 6-FAM and PI negative, R5 quadrant, Fig. 2iD) that accounted for 13.85% of all cell counts. For *Bacillus* sp. AU29, 6-FAM positive and PI negative (hybridized and likely living) cells (R3 quadrant, Fig. 2iiD) were detected at a frequency of 0.04%. 6-FAM and PI positive (hybridized, but dead) cells (R4 quadrant, Fig. 2iiD) accounted for 0.10% of all cell counts, which indicates that about 28% of the hybridized cells survived the procedure. The percentage of 6-FAM negative and PI positive (not hybridized and dead) cells (R6 quadrant, Fig. 2iiD) was higher (95.37%) than that for *Pseudovibrio* sp. SB55. The unstained (both 6-FAM and PI negative, R5 quadrant, Fig. 2iiD) population made up 4.49% of all cell counts.

Next, combinations of 1, 10 and 100 *Pseudovibrio* sp. SB55 cells were sorted into wells of 96-well plates for either the hybridized and alive (6-FAM positive and PI negative) or the non-hybridized and dead (6-FAM negative and PI positive) populations. For the 6-FAM positive and PI negative population, 4.17 ± 3.4% of the wells where 1 cell was added and 16.7 ± 7.8% of those with 10 cells showed turbidity, while no growth was observed in the wells containing 100 cells. For the 6-FAM negative and PI positive population, no turbidity was observed for any number of sorted cells (Supplementary Fig. S2). PCR and 16S rRNA gene sequencing confirmed the identity of *Pseudovibrio* sp. SB55 in all wells with growth.

For *Bacillus* sp. AU29, individual cells from the 6-FAM positive and PI negative (R3 quadrant, Fig. 2iiD), the 6-FAM and PI positive (R4 quadrant, Fig. 2iiD) as well as the 6-FAM negative and PI positive (R6 quadrant, Fig. 2iiD) populations were sorted into wells of 96-well plates. 6.25 ± 6% of wells containing cells from the 6-FAM positive and PI negative population showed turbidity, while the wells for the other two populations showed no growth (Supplementary Fig. S2). Again, PCR and 16S rRNA gene sequencing confirmed the identity of *Bacillus* sp. AU29 in all wells with growth. These results show that for both *Pseudovibrio* sp. SB55 and *Bacillus* sp. AU29 the counterstaining with PI allowed to select cells that are alive at the end of the live-FISH procedure and that hold a FISH signal.

### Sorting of specific cells from communities

The next step was to evaluate whether it was possible to selectively isolate a targeted bacterium from a mock community consisting of equal numbers of *Pseudovibrio* sp. SB55, *Bacillus* sp. AU29, and *Amphritea Atlantica* M41^T^. The mock community was sorted after live-FISH with the probe PARA739_6-FAM, which only targets *Pseudovibrio* sp. SB55. A clear 6-FAM-labelled cell population was visible (Fig. 2iiiA) when compared to the “non-sense” probe (Fig. 2iiiB) and “no probe” (Fig. 2iiiC) controls. Three populations were gated and sorted as single cells after counterstaining with PI: “6-FAM positive and PI negative” (hybridized and likely living cells - 0.03% of all event counts), “6-FAM negative and PI positive” (not hybridized and dead cells - 86.87%) and “6-FAM and PI negative” (unstained cells - 8.92%). The 6-FAM and PI positive (hybridized, but dead) cells accounted for 4.18% of all cell counts and were not sorted. This value indicates that about 0.7% of the hybridized cells within the mock community survived the procedure (Fig. 2iiiD).

5.6 ± 2.6% of the wells from the “6-FAM positive and PI negative” population showed turbidity and PCR and sequencing confirmed that all contained *Pseudovibrio* sp. SB55. In 1.4 ± 2% of the cases growth was observed in the wells containing cells from the “6-FAM negative and PI positive” population, which was, however, due to a contamination (*Staphylococcus epidermidi*s). Finally, 6.3 ± 5.1% of the wells from the unstained population (“6-FAM and PI negative”) showed turbidity and consisted of *Bacillus* sp. AU29 cells (Supplementary Fig. S3).

In order to sort labelled cells out of a complex natural community, *Pseudovibrio* sp. SB55 was inoculated into Baltic seawater and then the live-FISH procedure was applied. After FACS analysis it was also possible to detect a clear 6-FAM signal (Fig. 2ivA) when compared to the “non-sense” NONEUB338_6-FAM probe (Fig. 2ivB) and “no probe” (Fig. 2ivC) negative controls (R3 quadrant of the plots). Staining with PI allowed the detection and sorting of four different populations. Specifically, 6-FAM positive and PI negative (hybridized and likely living) cells made up 0.01% of all cell counts (Fig. 2ivD - R3 quadrant). 6-FAM and PI positive (hybridized, but dead) cells were detected at a frequency of 0.36%, which indicates that about 2.7% of the hybridized cells survived the procedure (Fig. 2ivD - R4 quadrant). The majority of cells (77.01%) were 6-FAM negative and PI positive and hence not hybridized and dead (Fig. 2ivD - gate between R5 and R6 quadrants). The unstained (both 6-FAM and PI negative) population accounted for 11.24% (Fig. 2ivD - R5 quadrant), while 11.38% consisted of all the other cells from the 6-FAM negative and PI positive population that were not sorted.

After sorting, turbidity was observed in 2.5 ± 0% of the wells containing individual 6-FAM positive and PI negative cells (R3) and the growth in these wells was confirmed to be *Pseudovibrio* sp. SB55. Growth was also observed in 3.13 ± 3% of the wells for the unstained population (R5) and consisted of contaminations with *S. epidermidis*. Growth was also found in 3.13 ± 3% of the wells for the 6-FAM and PI positive (R4) population and was was identified to be due to *Pseudovibrio* sp. SB55, probably due to cross-well contamination. Nothing grew where the 6-FAM negative and PI positive population was sorted (Supplementary Fig. S4).

### Application of live-FISH to an environmental sample

**Figure 3:**
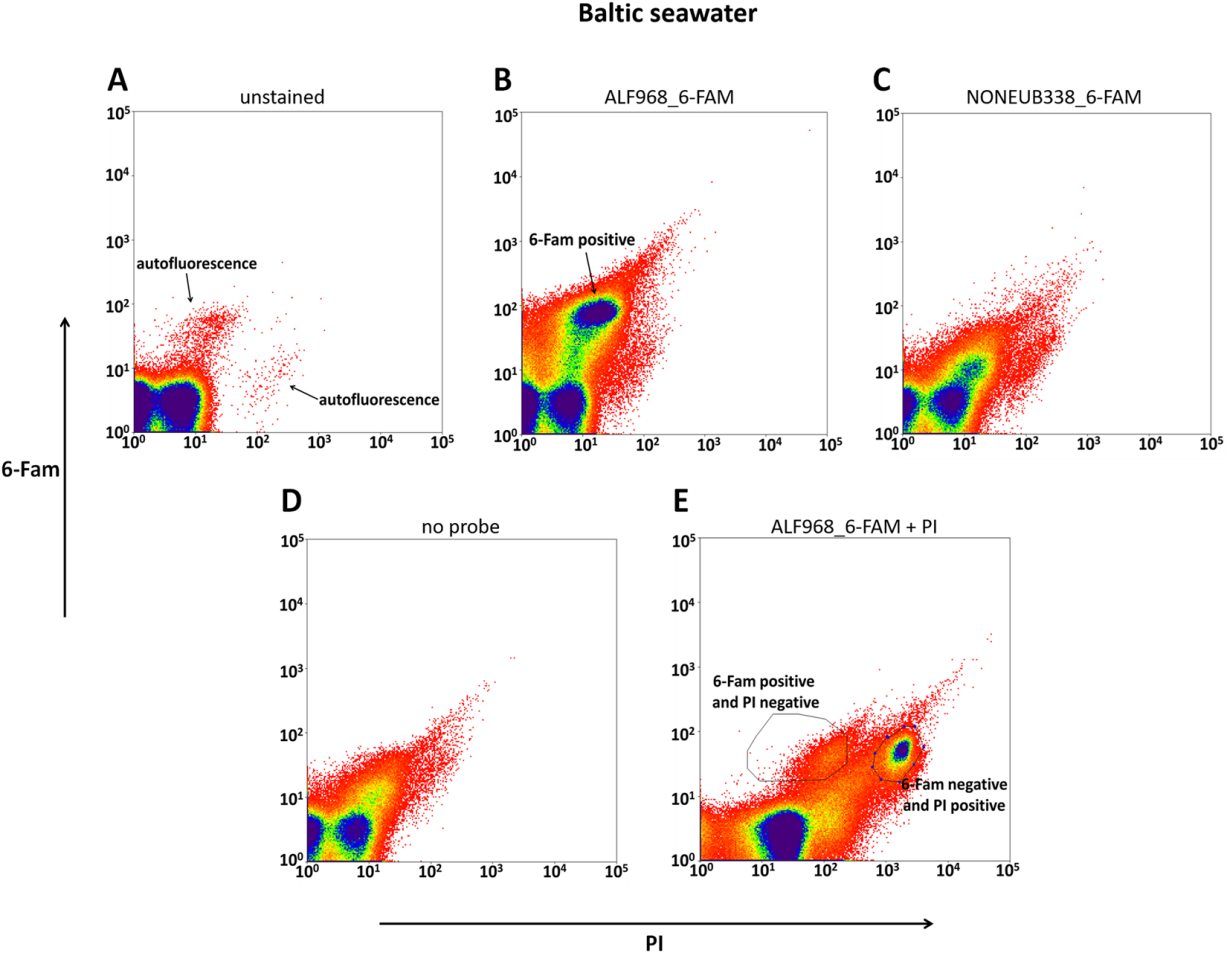
Signals for PI (X axes) and 6-FAM (Y axes) are plotted to show the sorting of Alphaproteobacteria from the Baltic seawater bacterial community after hybridization with the probe ALF968_6-FAM. (**A**) The arrows indicate the two populations of the unstained seawater bacterial community showing green and red autofluorescence. (**B**) The arrow points at the 6-FAM positive population after hybridization with the probe ALF968_6-FAM. (**C**) Negative control using the “non-sense” NONEUB338_6-FAM probe. (**D**) Negative control using “no probe” at all. (**E**) After counterstaining with PI, two populations were gated and sorted: 6-FAM positive and PI negative, and 6-FAM positive and PI positive.

The developed live-FISH method was finally applied directly to an environmental sample, and specifically to Baltic seawater, to test whether it was possible to target and isolate specific taxonomic members, such as Alphaproteobacteria, when hybridized with the ALF968_6-FAM probe. A clear 6-FAM signal (Fig. 3B) was detected when compared to the autofluorescence of the unstained seawater (Fig. 3A), and the negative controls using the “non-sense” NONEUB338_6-FAM probe (Fig. 3C) and “no probe” controls (Fig. 3D). After counterstaining with PI, two fluorescent populations were detected and sorted: 6-FAM positive and PI negative (1.77%) and 6-FAM positive and PI positive (6.09%) cells (Fig. 3E).

Ten cells from the two populations selected after counterstaining with PI (Fig. 3E) were sorted in 96-well plates (40 wells for each of the two populations per plate). 2.5% of the wells containing 6-FAM positive and PI negative (and hence living) cells showed turbidity indicating growing cells that were identified to be affiliated within the alphaproteobacterial genus *Brevundimonas*. No other taxon or contaminant was found in the cells of the sorted 6-FAM positive and PI negative population. Nothing grew in the wells corresponding to the 6-FAM negative and PI positive (and hence dead) population (Supplementary Fig. S5).

## Discussion

In this study, we developed a method called live-FISH to be used in combination with FACS to isolate living bacteria of interest solely based on their 16S rRNA gene sequence for subsequent culturing. We first showed that unfixed bacteria can survive the FFF procedure and that cell survival can be improved by optimizing factors, such as centrifugation speeds and resuspension buffers. Next, we demonstrated that labelled DNA probes can be introduced into living bacterial cells and provide evidence that specific hybridization with the 16S rRNA target occurs. Finally, we applied live-FISH to label specific target taxa within mock and natural microbial communities, which were then sorted by FACS and culture in a non-selective growth medium.

### Factors influencing viability of bacterial cells in the live-FISH procedure

Fixation is an important step for most quantitative FISH assays, because it preserves the integrity and shape of cells, while also permeabilizing them to allow for labelled oligonucleotides to diffuse to an intracellular rRNA target^40^. As membrane integrity is permanently compromised during fixation, cells are no longer viable^23^. Recent work has shown that fixation of cells is not necessary to obtain a FISH signal, however an ethanol treatment was still used to make cells permeable to the probe^18,19^. Here we show that replacing the ethanol treatment with a heat shock transformation allows for probe penetration, while keeping a small proportion of both Gram-positive and Gram-negative bacteria alive (Table 3). Critical steps to ensure high viability in live-FISH include centrifugation speeds and solutions for washing or resuspension, which is in line with previous observations^41,42^. For example, centrifugation at high speeds has been shown to directly diminish the culturability of *Escherichia coli* through cell loss, or indirectly impact viability of *Staphylococcus aureus* by altering the physicochemical properties of the cell surface^41,42^. Changing the washing or resuspension medium can also decrease the viability of *E. coli*, while different effects on the cell surface charge and hydrophobicity were observed for *S. aureus* and *Psychrobacter* sp. strain SW8 when high-salt solutions were replaced by low-salt buffers^41^. During a standard FISH protocol, cells are typically also exposed to the denaturant agent formamide in order to reduce cross-hybridization and ensure stringent reaction conditions^17^ as well as to the anionic surfactant SDS to increase probe accessibility and reduce clumping of excess probes during washing^43^. Formamide can impact cell integrity and this appears to depend on the thickness of the peptidoglycan layer^44^. Therefore, various formamide concentrations should be tested for any given target bacterium to achieve optimal hybridization, while minimising harmful effects on cell integrity^44^. SDS possesses strong biocidal properties by targeting the outer and cytoplasmic membranes as well as the membrane-bound enzymes^45^. This has led to questioning previous claims of hybridization in living bacteria in the presence of 0.05% SDS^21^, which probably killed the cells^23^. However, it is also known that individual cells within clonal bacterial populations exhibit marked phenotypic heterogeneity with variable degrees of resistance to antimicrobial treatments^46^, including SDS concentrations as high as 0.2%^47^ or 0.01%^48^. These observations are consistent with the minor loss of cell viability observed here when cells were exposed to 0.01% SDS (see pre-hybridization and hybridization steps in Table 4) in order to ensure viability, yet preventing probe clumping. Here, we further showed that the viability of the Gram-positive and Gram-negative bacterial strains tested significantly decreased with hybridization time and 2 h was chosen to allow probe hybridization, while minimizing bacterial cell loss (see Table 4). This is also in line with the minimum hybridization time recommended to observe a specific signal at probe concentrations from 1 to 5 ng µl^−1^ during the standard FISH procedure^49^.

### Probe transfer into the cell

One obvious problem when working with unfixed and thus not permeable cells is the delivery of DNA into the cytoplasm. Fluorescently labelled oligonucleotide do not readily pass the cell membrane^24^. Over the past decades, a number of techniques have been developed to deliver RNA-targeting fluorescent probes into living cells, including microinjection as well as treatment with polycationic molecules (such as liposomes and dendrimers), cell-penetrating peptides and streptolysin O^24^. The application of these techniques is however limited to specific eukaryotic cell types^50^. Here we showed by means of microscopy (see Fig. 1) and FACS (see Figs. 2 and 3) that it is possible to transport fluorescently labelled oligonucleotides into living cells through a heat shock transformation. Heat shock transformation is generally used to introduce large, non-labelled DNA molecules (e.g. plasmid) into living bacteria cells. Transformation is based on creating a calcium- and/or magnesium-rich environment to counteract the electrostatic repulsion between DNA and the bacterial cellular membrane and by a sudden increase in temperature that transiently creates pores in the plasma membrane^34^. In a standard transformation procedure, the heat shock is followed by a “recovery step”, where cells are immediately incubated in a rich medium to restore their plasma membrane^20^. However, experiments have shown that, for example, *E. coli* cells are able to be resuscitated even after being subjected to 55 °C for 1 h or to light-induced oxidative stress for up to 8 h^51^. This means that cells are able to survive extended periods of stress and still divide again under more favourable conditions, which would be consistent with the recovery of viable cells seen in our live-FISH protocol, where an immediate recovery in media was omitted.

Heat shock has often been used to introduce plasmid DNA into a wide range of bacteria and Euryarchaeota, including *E. coli*, *Pseudomonas aeruginosa*, *Salmonella typhimurium* and *Halobacterium salinarum*^20,32,33,52,53^. Transformation efficiencies range typically from 10^6^ to 10^9^ CFU µg^−1^ of supercoiled plasmid and typically 0.001 to 1% of cells are being transformed^33^. These percentages of transformed cells are broadly in line with the percentages of the living and hybridized cells measured after counterstaining with PI for *Pseudovibrio* sp. SB55 (see Fig. 2i) and *Bacillus* sp. AU29 (see Fig. 2ii). The success of heat shock transformation is generally considered to be limited by the nature of the cell wall, which is thicker in Gram-positive bacteria^20^. However, we found similar percentages of living and hybridized *Pseudovibrio* sp. SB55 and *Bacillus* sp. AU29 cells (see Fig. 2i and ii), which could perhaps be explained by the natural competence to take up exogenous DNA for members of the genus *Bacillus*^54^ or by the possibility that the cell wall might represent less of a barrier for small oligonucleotides compared to large plasmid molecules.

### Hybridization of probes in living cells

Even after a oligonucleotide probe successfully enters a cell, only a fraction of the probe will likely hybridize to intracellular rRNA^24^. Standard DNA probes are prone to degradation by cellular nucleases^55^. Probes with modified nucleotides, such as 2′-O-methylated oligonucleotides, have been shown to have increased nuclease stability, which makes them especially suited for hybridization of living cells^56^ and could thus be used in the future to increase cellular probe availability in our protocol. In addition, the access of oligonucleotide probes to their target site may be impaired by the three-dimensional structure of the ribosome, which includes rRNA-rRNA interactions as well as interactions with ribosomal proteins^57–59^. However, in metabolically active bacterial cells, rRNA genes are also constantly transcribed^60^ and these transcripts could be directly hybridized with a probe before being incorporated into the ribosome^60^. In fact, free mRNA has recently been shown to be a suitable target for probe hybridization^61–64^.

We demonstrated here that hybridization is specific in the live-FISH process, as the two strains tested (*Pseudovibrio* sp. SB55 and *Bacillus* sp. AU29) showed specific fluorescent signals when compared to the “non-target” controls. These controls consisted of treating *Pseudovibrio* sp. SB55 cells with the probe for *Bacillus* sp. AU29, and vice versa (see Fig. 2i and ii), which resulted in more than three mismatches. Silverman and Kool^21^ were previously able to discriminate living cells of *E. coli* and *Salmonella enterica* with specific quenched autoligation (QUAL) probes that differed by a single nucleotide. This approach is however limited by the high cost and the design restriction of QUAL probes (e.g. there needs to be a specific nucleotide in positions where the fluorophore and the quencher are attached). It was also later questioned by Amann and Fuchs^23^ whether Silverman and Kool^21^ had truly shown hybridization of living cells, since they found by live/dead staining that the hybridized *E. coli* cell suspension comprised mainly of dead cells and that cultivation was not possible. In our study, we were however able to sort and subsequently culture living cells after live-FISH (see Fig. 2i and ii and Supplementary Fig. S2). This makes our work, to the best of our knowledge, the first successful demonstration of probe hybridization and sorting of living bacterial cells, with other examples only existing to date for eukaryotic cells^24,62,63^. For example, dual-labelled oligonucleotides were used to specifically label cardiomyocytes (CMs) differentiated from mouse or human pluripotent stem cells and sort living cells with FACS to approximately 97% purity as confirmed by electrophysiology and immunocytochemistry^62^. Using a similar approach, fluorescent DNA probes were used to purify working-type and living CMs from nodal-type CMs^63^.

### Future developments for live-FISH

When applying the live-FISH procedure and combination with FACS to artificial and natural microbial communities, we found support for the specificity of the method in targeting taxa of interest purely based on their sequence information and fluorescent signal. To the best of our knowledge, there are currently no other methods that can isolate specific bacteria from environmental samples purely based on targeting their 16S rRNA gene sequence. A number of single-cell techniques (e.g. Raman-activated cell sorting or microfluidics) are currently being developed to sort specific cell types based on morphological or physiological properties^65,66^, and they could be combined with live-FISH to contribute to the cultivation of yet-uncultivable microorganisms. To achieve this goal, the formulation of new cultivation media based on multi-omics information^67–70^ should be also considered.

Some limitations of the live-FISH procedure developed here are the sorting efficiency, the risk of contaminations and the type of bacteria and environmental samples to work with. This should be subject to future considerations, developments and improvements.

Sorting efficiency is defined as the number of target cells sorted to the total number of target cells detected and is dependent on (1) the sort mode chosen and (2) the set-up of the sorting system^71^. When working with single cells, it is crucial to verify whether only a single target cell is contained in every droplet that is going to be sorted. Sorting of higher numbers of cells could enhance the chances of targeting the cells of interest, but also the risk of haphazardly sorting non-target cells^71^. FACS sorters are usually calibrated in order to check for the proper alignment of the droplets to the wells of a multi-well plate. However, calibration can fail and inert dyes have recently been used in sheath fluids to quickly tell how many wells have been sorted correctly^72^. Our results showed that the growth success in the wells was to a certain extent independent of the number of cells sorted in each well, thus pointing to the possibility that the droplet had not successfully reached the medium in the bottom of the well. This could in the future be checked with such inert dyes^72^. Several studies have also reported sorting efficiencies of the targeted populations typically exceeding 95%^73–75^. However, it is also known that different types of bacteria exhibit different survival rates in the sorting procedure^76^. We found that the bacteria tested here showed a survival rate of approximately 50% when incubated in the sheath fluid used for sorting, and this could negatively impact on the sorting efficiency observed in this study. In the future, a range of different sheath fluids could be tested to ensure maximum viability.

Another common problem of sorting large numbers of events from complex systems is the potential for sorting contaminants, which can come from the instrument, reagents or materials as well as haphazard sorting of non-target cells^77^. The contaminations observed here (i.e. *S. epidermidis*) were however more likely due to plate handling or to cross-well contamination during incubation. These issues are often observed in FACS sorting, especially when working with single cells^19^, and could be overcome by using sorters enclosed in sterile benches. Experimental reagents are another recognized source of contamination^78^, and could be checked in the future by checking single, cell-free drops of the sheath fluid into media.

The application of live-FISH directly to an environmental sample confirmed that it was possible to specifically target and isolate taxonomic groups of interest, e.g. Alphaproteobacteria, as represented by the genus *Brevundimonas*. Bacteria belonging to this genus have previously been found in Baltic seawater using both culture-independent and culturing approaches^79,80^. However, there are some factors that could limit our approach when applied to different types of environmental samples. As live-FISH is based on fluorescence, samples with high autofluorescence in certain wavelengths will require a choice of a probe fluorophore outside of this range^19^. This could be overcome by using fluorophores in the ultraviolet (UV) and infrared (IR) ranges of the electromagnetic spectrum^81–83^. The number of ribosomes, and hence available target sites, is often also affected by environmental conditions (e.g. light exposure, temperature, pH and oxygen rate, to name a few)^84–86^ and this can lower the FISH signal for environmental microorganisms compared to pure cultures grown under optimal conditions^73,87^. However, when *Pseudovibrio* sp. SB55 was inoculated in seawater and thus likely nutrient-limited and not actively dividing anymore, we could still detect a specific fluorescent signal and distinguish it from the rest of the seawater’s microbial community (see Fig. 2iv). When we applied live-FISH directly to the seawater, the fluorescent signal for Alphaproteobacteria was however less intense (see Fig. 3B), indicating some limitation of probe binding. There have been attempts to improve signal detection by boosting cellular ribosome content before fixation during a standard FISH procedure^88^, for example, by pre-incubating samples in a cocktail of nutrients and antibiotics, which should in theory result in cell activation and rRNA synthesis without cell division. In fact, improved fluorescent signals were reported for an oligotrophic cooling water system after pre-treatment with glucose and chloramphenicol^89^. The problem with this approach is however the inevitable selectivity of substrates and antibiotics^88^. It is also known that the physiological history of the cell can influence its ribosome content^90^. It has been shown that, for example, some slow-growing chemolithoautotrophic bacteria still possess high cellular rRNA concentrations and are therefore detectable by standard FISH protocols, even after extended periods of complete physiological inhibition or starvation^91–93^. In contrast, bacterial cells might be highly active despite a low ribosome content and low FISH detectability, as demonstrated for the SAR86 lineage in marine samples^94^. This means that the sensitivity of the fluorescent signal using our protocol, as for standard FISH application, could be dependent on the type of sample and bacteria under analysis. One way to boost sensitivity and overcome this limitation could be the use of multi-labelled probes as was recently employed to identify unfixed microbial symbionts in the gutless oligochaete *Inanidrilus leukodermatus*^95^.

## Conclusions

The results of this study show that live-FISH, when used in combination with FACS, allows for the targeted isolation and cultivation of Gram-positive and Gram-negative living bacteria that belong to certain taxonomic groups as defined by a probe. We believe that our method can not only be used for the isolation of living bacteria of interest by specifically labelling and sorting them into non-selective media, but also offers a great potential for the culturing of new microorganisms in parallel or in combination with the design of new and specific cultivation media and methods. We therefore think that our method will have the potential to enhance the culturing success in a range of system, including from the microbiota found in humans, animals, plants, soils and aquatic environments.

## Supplementary information

Supplementary Info
