# Supplementary material for: Fluorescence in situ hybridization (FISH) and cell sorting of living bacteria
Source: Sci Rep. 2019 Dec 9;9:18618. doi: 10.1038/s41598-019-55049-2 (PMC6901588; doi:10.1038/s41598-019-55049-2)
Supplement: Supplementary file 1 — Supplementary Info [file 41598_2019_55049_MOESM1_ESM.docx]

**Supplementary Information**

**Fluorescence *in situ* hybridization (FISH) and cell sorting of living bacteria**

Giampiero Batani^1,2^, Kristina Bayer^3^, Julia Böge^3^, Ute Hentschel^3,4^ and Torsten Thomas^1*^

^1^Centre for Marine Science and Innovation and School of Biological, Earth and Environmental Sciences, The University of New South Wales, Sydney, New South Wales, Australia

^2^Faculty of Science, University of South Bohemia, Ceske Budejovice, Czech Republic

^3^GEOMAR Helmholtz Centre for Ocean Research Kiel, Düsternbrooker Weg 20, 24105 Kiel, Germany

^4^Christian-Albrechts University of Kiel, Christian-Albrechts-Platz 4, 24118 Kiel, Germany

*Corresponding author: email: t.thomas@unsw.edu.au

**Supplementary Figures**


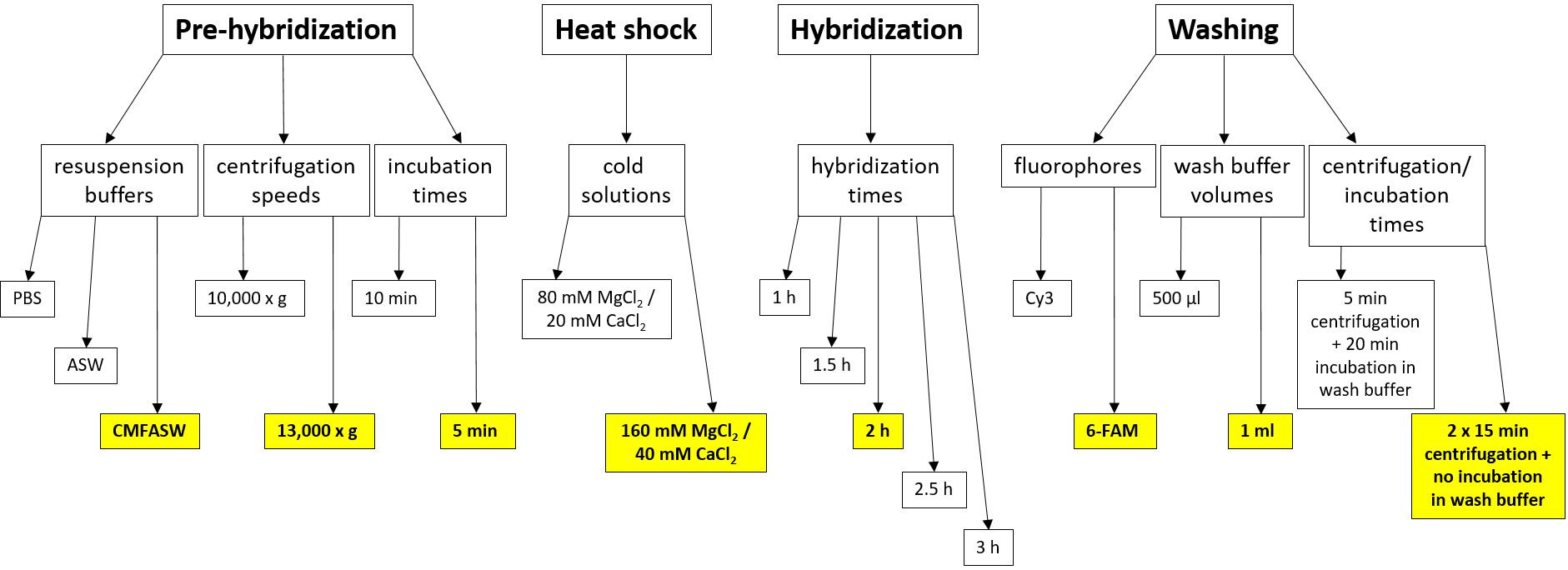


**Supplementary Figure S1.** Optimization processes undertaken at each FISH step. The yellow boxes show the conditions used in the final live-FISH protocol (see Table 2) that maximized viability of both Gram-positive and Gram-negative bacteria. PBS= phosphate Buffered Saline solution; ASW=artificial seawater; CMFASW= Ca^2+-^ and Mg^2+-^free artificial seawater.

**Supplementary Figure S2.** Optimization of the hybridization step. **a)** CFU ml^-1^ is expressed as a function of time (1, 1.5, 2, 2.5 and 3 h of incubation) for both *Bacillus* sp. AU29 and *Ruegeria* sp. AU82. Error bars show standard deviations. **b)** The fluorescence images show how a signal in the cells was only observed from 2 h of hybridization onwards for both species analyzed. Scale bars, 10 µm.

**Supplementary Figure S3.** Growth of (A) *Pseudovibrio* sp. SB55 hybridized with the probe PARA739_6-Fam and (B) *Bacillus* sp. AU29 hybridized with the probe LGC339_6-Fam, and both counterstained with PI. In panel (A), arrows point at the wells showing turbidity and containing 1 and 10 cells from the 6-Fam positive and PI negative (and hence potentially live) population. The identity of *Pseudovibrio* sp. SB55 was confirmed in all wells with growth. Nothing grew in the wells where 100 cells of the 6-Fam positive and PI negative population and from the 6-Fam negative and PI positive (and hence dead) population were sorted (see Fig. 2 i.). In panel (B), the arrows point at the wells showing turbidity in the R3 population (6-Fam positive and PI negative) and corresponding to *Bacillus* sp. AU29, while no growth was observed for the R4 (6-Fam positive and PI positive) and R6 (6-Fam negative and PI positive) populations (see Fig. 2 ii.). The control wells with only marine broth (MB - e.g. where no cells were sorted into) were negative.

**Supplementary Figure S4.** Growth of *Pseudovibrio* sp. SB55 within the mock community hybridized with the probe PARA739_6-Fam and counterstained with PI. The arrows point at the wells showing turbidity in the three populations gated and sorted (see Fig. 2 iii.). In the “6-Fam positive and PI negative” (and hence potentially live *Pseudovibrio* sp. SB55) population, growth corresponded successfully to *Pseudovibrio* sp. SB55 cells. In the “6-Fam negative and PI positive” (and hence dead) population, turbidity corresponded to contamination by *Staphylococcus epidermidis*. Finally, in the “6-Fam negative and PI negative” population corresponding to all unstained events, growth consisted of *Bacillus* sp. AU29 cells. The control wells with only marine broth (MB - e.g. where no cells were sorted into) were negative.

**
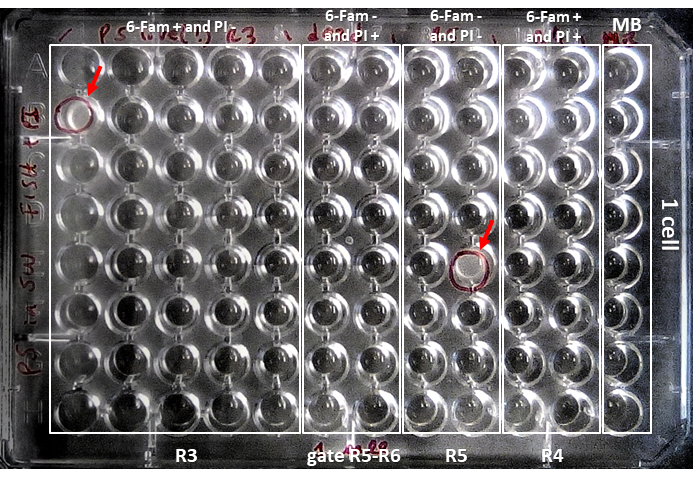
**

**Supplementary Figure S5.** Growth of *Pseudovibrio* sp. SB55 inoculated into Baltic seawater, hybridized with the probe PARA739_6-Fam and counterstained with PI. Turbidity (arrows) was observed only in the 6-Fam positive and PI negative (and hence potentially live) population (R3) and in the 6-Fam negative and PI negative (R5) one corresponding to all unstained events (see Fig. 2 iv.). Growth in the 6-Fam positive and PI negative population corresponded to *Pseudovibrio* sp. SB55, while turbidity in the 6-Fam negative and PI negative one was due to contamination. The control wells with only marine broth (MB - e.g. where no cells were sorted into) were negative.

**
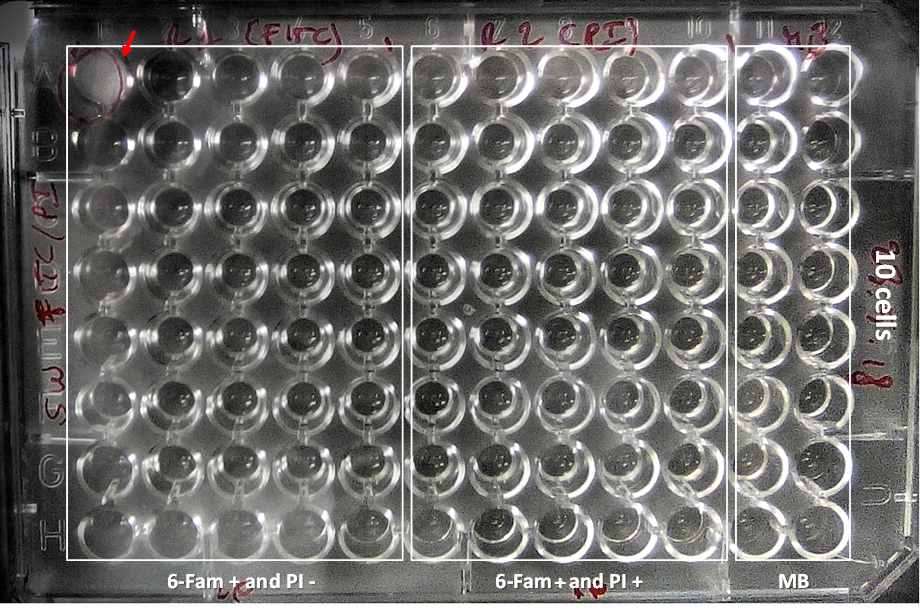
**

**Supplementary Figure S6.** Growth of Alphaproteobacteria from the Baltic seawater microbial community when hybridized with the probe ALF968_6-Fam. The arrow points at the well showing turbidity in the 6-Fam positive and PI negative population (corresponding to potentially living cells - see Fig. 3) and confirmed to be affiliated within the alphaproteobacterial genus *Brevundimonas*. Nothing grew in the wells corresponding to the 6-Fam positive and PI positive (and hence dead) population (see Fig. 3) and the control ones with only marine broth (MB - e.g. where no cells were sorted into).

**Supplementary Tables**

**Supplementary Table S1.** Comparison of the cell viability between each of the eleven treatments (combinations of different buffers, centrifugation speeds and incubation times during the pre-hybridization step of the live-FISH procedure) and the conditions of the modified FFF protocol (PBS - 10,000 x g - 5 min) for *Bacillus* sp. AU29 and *Ruegeria* sp. AU82. Significance was assessed with a Kruskal-Wallis test.

*ns = not significant;

*mean rank differences = differences between the mean rank of the modified FFF protocol and the mean rank of each treatment. *Boldface indicates statistical significance (p ≤ 0.05 for the Kruskal-Wallis test, and *p ≤ 0.05 but > 0.01 and **p ≤ 0.01 for the Dunn’s multiple comparisons test).

**Supplementary Table S2.** Comparison of the cell viability between each of the twelve treatments (combinations of different buffers, centrifugation speeds and incubation times during the pre-hybridization step of the live-FISH procedure) and the control consisting of cell numbers before the treatments (e.g. after incubation overnight) for both *Bacillus* sp. AU29 and *Ruegeria* sp. AU82. Significance was assessed with a Kruskal-Wallis test.

*ns = not significant;

*mean rank differences = differences between the mean rank of the control and the mean rank of each treatment.

*Boldface indicates statistical significance (p ≤ 0.05 for the Kruskal-Wallis test, and *p ≤ 0.05 but > 0.01 and **p ≤ 0.01 for the Dunn’s multiple comparisons test).

**Supplementary Table S3.** Comparison of the cell viability between each of the three treatments (combination of incubations in different cold solutions during the heat shock step of the live-FISH procedure) and the control consisting of cell numbers before the treatments (e.g. after pre-hybridization) were tested on both *Bacillus* sp. AU29 and *Pseudovibrio* sp. SB55. Significance was assessed with a Kruskal-Wallis test.

*ns = not significant;

*mean rank differences = differences between the mean rank of the control and the mean rank of each treatment.

*Boldface indicates statistical significance (p ≤ 0.05 for the Kruskal-Wallis test, and *p ≤ 0.05 but > 0.01 for the Dunn’s multiple comparisons test).
